# RAD51B plays an essential role during somatic and meiotic recombination in Physcomitrella

**DOI:** 10.1093/nar/gku890

**Published:** 2014-09-26

**Authors:** Florence Charlot, Liudmila Chelysheva, Yasuko Kamisugi, Nathalie Vrielynck, Anouchka Guyon, Aline Epert, Sylvia Le Guin, Didier G. Schaefer, Andrew C. Cuming, Mathilde Grelon, Fabien Nogué

**Affiliations:** 1 INRA, Institut Jean-Pierre Bourgin UMR1318, Saclay Plant Sciences, Versailles, France; 2 AgroParisTech, Institut Jean-Pierre Bourgin UMR1318, Saclay Plant Sciences, Versailles, France; 3 Centre for Plant Sciences, Faculty of Biological Sciences, Leeds University, Leeds LS2 9JT, UK; 4 Laboratoire de Biologie Moleculaire et Cellulaire, Institut de Biologie, Universite de Neuchatel, rue Emile-Argand 11, CH-2007 Neuchatel, Switzerland

## Abstract

The eukaryotic RecA homologue Rad51 is a key factor in homologous recombination and recombinational repair. Rad51-like proteins have been identified in yeast (Rad55, Rad57 and Dmc1), plants and vertebrates (RAD51B, RAD51C, RAD51D, XRCC2, XRCC3 and DMC1). RAD51 and DMC1 are the strand-exchange proteins forming a nucleofilament for strand invasion, however, the function of the paralogues in the process of homologous recombination is less clear. In yeast the two Rad51 paralogues, Rad55 and Rad57, have been shown to be involved in somatic and meiotic HR and they are essential to the formation of the Rad51/DNA nucleofilament counterbalancing the anti-recombinase activity of the SRS2 helicase. Here, we examined the role of RAD51B in the model bryophyte *Physcomitrella patens*. Mutant analysis shows that RAD51B is essential for the maintenance of genome integrity, for resistance to DNA damaging agents and for gene targeting. Furthermore, we set up methods to investigate meiosis in Physcomitrella and we demonstrate that the RAD51B protein is essential for meiotic homologous recombination. Finally, we show that all these functions are independent of the SRS2 anti-recombinase protein, which is in striking contrast to what is found in budding yeast where the RAD51 paralogues are fully dependent on the SRS2 anti-recombinase function.

## INTRODUCTION

DNA double-strand breaks (DSBs) are a major threat for genome integrity and the accumulation of such damage leads to oncogenesis, cell death and severe dysfunction of cells. The DNA DSB repair system is crucially important for survival of living cells and highly conserved in eukaryotes. DSB repair can be achieved by multiple pathways, which can be grouped into two classes based on the involvement of sequence homology: non-homologous end-joining (NHEJ) and homologous recombination (HR). Repair of DSBs through HR generally preserves the integrity of the genomic material. In addition, HR events play an essential role in assuring proper meiotic chromosomal disjunction and represent an essential mechanism to create genetic diversity.

The RECA/RAD51 family proteins are involved in HR mechanisms in prokaryotic and eukaryotic cells and have been the object of considerable interest (for review see (1)). In the budding yeast *Saccharomyces cerevisiae*, 4 RecA-like homologues, Rad51, Rad55, Rad57 and Dmc1, have been identified. *Rad51*, *Rad55* and *Rad57* are expressed ubiquitously while *Dmc1* is a meiosis-specific gene. *Rad51* and *Dmc1* genes are conserved in many eukaryotes, while *Rad55* and *Rad57* seem to be specific to yeasts. All these Rad51-like proteins play roles in DSB repair including HR (2) and yeast mutants impaired in any of the Rad51-like proteins are defective in meiotic recombination and exhibit reduced spore viability (3). Even though Rad51 and Dmc1 have been demonstrated to be the core protein for the formation of the nucleoprotein filament for strand invasion, the function of the Rad55 and Rad57 paralogues in the process of HR is less clear. However, a recent study has demonstrated that the Rad55/Rad57 complex is involved in the formation of the Rad51/DNA nucleofilament, showing that this complex acts to counterbalance the anti-recombinase activity of SRS2 helicase (4).

In addition to RAD51 and DMC1, five RAD51 paralogues (XRCC2, XRCC3, RAD51B/RAD51L1, RAD51C/RAD51L2 and RAD51D/RAD51L3) have been identified in mammals, with 20–30% protein sequence identity with RAD51 and with each other (5). Unlike RAD51, no self-assembly of individual paralogues has been detected. Biochemical studies have unravelled different complexes: RAD51B-RAD51C-RAD51D-XRCC2, RAD51C-XRCC3, RAD51C-RAD51B, RAD51C-RAD51D and RAD51C-RAD51D-XRCC2 (reviewed in (6)). The functions of these various subcomplexes are still unclear mostly because knockouts of the RAD51 paralogue genes are embryo-lethal in animal model systems and hardly any *in vivo* data are available (7–11). Nevertheless, RAD51 paralogue functions investigated using immortalized vertebrate cell lines, such as chicken DT-40 or hamster CHO cells (12–15), showed that RAD51 paralogues all play roles in somatic recombination, DNA repair and chromosome stability (for reviews see (5)). One of the complexes, the RAD51B/RAD51C complex, has been shown to promote strand exchange activity of RAD51-ssDNA filaments (16,17). Based on *in vitro* studies it has been recently proposed that this function could be mediated by the direct formation of the RAD51B/RAD51C complex on ssDNA that would partially stabilize the RAD51 nucleoprotein filament against the anti-recombinogenic activity of the Bloom's syndrome (BLM) helicase (18). Consistent with a major role of RAD51B in HR, chicken DT40 *rad51b* mutant cell lines show spontaneous chromosomal aberrations, high sensitivity to cross-linking agents, mild sensitivity to gamma rays and significantly attenuated RAD51 focus formation after gamma ray exposure. In addition, RAD51B deficiency abolishes targeted integration in the Chicken DT40 cell line mutant (14). Finally, the high expression levels of RAD51B observed in testis (19) suggest that this protein could also be involved in animal meiosis and possibly in meiotic HR.

The five RAD51 paralogues described in vertebrates, including RAD51B, have been identified in plants (20) and corresponding mutants have been characterized (21–34). Analysis of mutants affected in *RAD51B* supports a conservation of functions of this paralogue between animals and plants. Indeed, disruption of the *RAD51B* gene in *Arabidopsis thaliana* (Arabidopsis) confers hypersensitivity to the DNA cross-linking agents mitomycin C and cisplatin (21,22) and leads to hypersensitivity to bleomycin when combined with disruption of the *XRCC2* and/or *RAD51D* genes (23). Moreover, spontaneous somatic recombination was shown to be reduced in the Arabidopsis *rad51b* mutant (24,35). The somatic role of the plant RAD51B paralogue contrasts with its minor role during meiotic HR. Indeed, the Arabidopsis *rad51b* mutant is fully fertile (21,22) and shows only a very weak increase in meiotic recombination rates (24).

Due to their tolerance, in term of development, to mutation of the RAD51 paralogues, plants represent a very attractive model to study the role of these proteins during different phases of development, and particularly meiosis. In the plant kingdom, the moss *Physcomitrella patens* (Physcomitrella) exhibits rates of gene targeting (GT) comparable to *S*. *cerevisiae* and has permitted advances in the comprehension of HR-mediated DNA repair and transgene integration via GT in plants (36–42). To gain further knowledge on somatic and meiotic HR in plants, we investigated the role of RAD51B in somatic and meiotic recombination. The results show that RAD51B is indispensable for DNA damage repair and HR in somatic cells and that this role is independent of the SRS2 anti-recombination helicase. Moreover, we show that if some RAD51 functions are clearly dependent on RAD51B, others, like its role in development of Physcomitrella, are RAD51B independent. Finally, our data show for the first time an essential role for RAD51B during meiosis.

## MATERIALS AND METHODS

### Plant material

*Physcomitrella patens* (Hedw.) B.S.G. ‘Gransden2004’ was vegetatively propagated as previously described (43). Individual plants were cultured as ‘spot inocula’ on BCD agar medium supplemented with 1 mM CaCl2 and 5 mM ammonium tartrate (BCDAT medium), or as lawns of protonemal filaments by subculture of homogenized tissue on BCDAT agar medium overlaid with cellophane for the isolation of protoplasts. Transformation experiments were performed as previously described (36) using linear fragments of DNA generated by digestion of transforming vectors with restriction enzymes (40). Growth conditions for the generation of deletion strains were performed as described (41). The *rad51-1-2* double mutant has been described previously (41).

### Gene identification and isolation

Genomic DNA and total RNA was isolated from Physcomitrella as previously described. For verification of gene models, RNA was extracted as described in (44). Physcomitrella genomic sequences encoding the *RAD51B* and *SRS2* genes were identified by Basic Local Alignment Search Tool (http://www.phytozome.net/Physcomitrella_er.php). The available gene models were used for the design of polymerase chain reaction (PCR) primers to amplify cognate genomic sequences, which were cloned in the TOPO-TA (Life Technologies, USA) or pBluescript (Stratagene, USA) plasmids. PCR primers used are listed in Supplementary Table S1. In order to obtain a correct gene model for each sequence, full-length cDNA sequences were amplified from Physcomitrella polyribosome-derived RNA by rt-PCR (44). Predicted polypeptide sequences were aligned with the orthologous genes from Arabidopsis and *Homo sapiens* using CLUSTALW.

### Targeted gene knockout

For generation of deletion mutant *rad51BΔ*, the 5′- and 3′-targeting fragments were amplified from Physcomitrella genomic DNA and cloned upstream (5′) and downstream (3′) of the loxP sites flanking the resistance cassette in plasmid pBNRF (41) to create pRAD51Bdelta. For the deletion mutant *srs2Δ*, the 5′- and 3′-targeting fragments were amplified from Physcomitrella genomic DNA and cloned on either side of the *Sal*I site of plasmid pUC18. A loxP-flanked G418 resistance cassette from pMBL5DL (44) was cloned into this site to create pSRS2delta. For the *PpRAD51B* gene, a 475 bp 5′-targeting fragment (coordinates 11046726-11046252 on chromosome 25) and a 891 bp 3′-targeting fragment (coordinates 11042979–11042088 on chromosome 25) were PCR-amplified. For *PpSRS2*, a 607 bp 5′-targeting fragment (coordinates 20773717-20774323 on chromosome 1) and a 1043 bp 3′-targeting fragment (coordinates 20783155–20784197 on chromosome 1) were PCR-amplified.

Moss protoplasts were transformed with pRAD51Bdelta digested with *Avr*II and *BsrG*I, or with a 3.2 kb pSRS2delta targeting fragment obtained by PCR amplification. Stable disruptants were selected by successive subculture on selective and non-selective medium and PCR analysed as described in (44). Clean deletions in the *PpRAD51B* (exons 3–10) and *PpSRS2* (exons 2–24) genes were obtained by transient Cre recombinase expression (40). Deletions in the recombinant loci were confirmed by PCR amplification for five independent clones using gene-specific external primers PpRAD51B#1 and PpRAD51B#2, and PpSRS2#10 and PpSRS2#11, respectively. Primers APT#16 and APT#19 were used as positive controls (Supplemental Table S1). The five *rad51bΔ* deletion mutants show similar phenotypes when tested. Two out of the five independent clones are described in this study.

### Targeted gene knockin

In order to replace the *PpRAD51B* gene from ATG to stop by the *AtRAD51B* cds, we produced the pPpAtRAD51B-KanR plasmid. First, we amplified a PCR fragment covering the *AtRAD51B* cDNA region (21) using Arabidopsis cDNA as the starting template (coordinate 47-1037 on Genbank: AB194809) and cloned it into a pUC18 plasmid to obtain pAtRAD51B. To generate the hybrid Physcomitrella-Arabidopsis *RAD51B* expression cassette pPpAtRAD51B, a 575 bp 5′ targeting fragment (partial promoter/5′UTR of *PpRAD51B*, coordinates 11047187-11046613 on chromosome 25) and a 560 bp 3′ targeting fragment (partial 3′UTR of *PpRAD51B*, coordinate 11042647-11042088 on chromosome 25) were amplified from Physcomitrella genomic DNA and cloned in pPpAtRAD51B upstream of the ATG and downstream of the stop codon of the *AtRAD51B* cDNA, respectively. To obtain pPpAtRAD51B-KanR, the 35S::neoR cassette flanked by two LoxP sites was recovered from pBNRF (41) and inserted in pPpAtRAD51-2 between the *AtRAD51B* cDNA stop codon and the 3′UTR of *PpRAD51B*.

Moss protoplasts were transformed with pPpAtRAD51B-KanR digested with *BsrG*I. Stable disruptants were selected by successive subculture on selective and non-selective medium and PCR analysed as described in (44). Elimination of the 35S::neoR cassette was obtained by transient Cre recombinase expression (40). Replacement of the *PpRAD51B* gene by the *AtRAD51B* cds at the recombinant loci was confirmed by PCR amplification using locus-specific external primers PpRAD51B#1 and PpRAD51B#2. Primers APT#16 and APT#19 were used as positive controls (Supplementary Table S1).

### Analysis of gene expression in mutants

Transcript abundance in selected knockout and knockin lines was determined by rt-PCR of cDNA as described in (44). Detection of *RAD51B* and *SRS2* mRNA in mutant lines was by rt-PCR using primers indicated in Supplementary Table S1.

### Bleomycin and ultraviolet (UV)-B sensitivity assays

Protoplasts of wild-type (WT), *rad51B* and *rad51-*1-2 mutants in BCDAT liquid medium supplemented with mannitol were treated with bleomycin (Bleocin inj., Euro Nippon Kayaku GmbH, Germany) at concentrations indicated in the text for 1 h. Protoplasts were washed two times and then resuspended in liquid mannitol medium. After 20 h in the dark, the protoplasts were spread on BCDAT agar medium supplemented with mannitol (ca. 10^5^ protoplasts per Petri dish). After 6 days regeneration, the number of survivors was counted. We repeated these experiments three times.

Protoplasts of WT, *rad51B* and *rad51-*1-2 mutants were spread (ca. 50 000/plate) on protoplast agar medium (PpNH4 + 0.5% glucose + 8.5% mannitol). Plates were immediately irradiated with UV-B light (308 nm) in a Stratagene Stratalinker. The intensity of the irradiation was controlled using the internal probe of the Stratalinker and one plate of each strain was treated simultaneously. The experiment was repeated three times. Plates were immediately transferred to darkness for 24 h after treatment then to standard growth conditions for protoplast regeneration. Survival was determined after 1 week by microscopic observation.

### Evaluation of spontaneous mutation frequency

Mutations in the *PpAPT* gene confer resistance to 2-fluoroadenine (2-FA), a toxic compound for cells. The number of 2-FA resistant colonies that grow following protoplast regeneration reflects the frequency of spontaneous mutations. Protoplasts of WT and *rad51b* mutant were regenerated for 6 days on BCD agar medium supplemented with mannitol (ca. 10^5^ protoplasts per Petri dish) and then transferred on to BCDAT agar medium supplemented with 10 μM 2-FA (Fluorochem). After 2 weeks, the number of resistant colonies was counted. Experiments were repeated three times and statistically analysed using the Fisher's exact test. The adenine phosphoribosyl transferase (*APT*) genomic sequence from 2-FA resistant clones was subsequently PCR amplified using primers PpAPT#2 and PpAPT#20 (Supplementary Table S1) and sequenced to identify the mutation responsible for the resistance.

### GT assays

Transformation efficiency and *APT* targeting frequency were measured as previously described (41). Moss protoplasts (4.8 × 10^5^) were transformed with the non-homologous pBHRF plasmid (41) digested with *Hind*III to produce a linear fragment containing the 35S::hygroR marker, or with the PpAPT-KO2 plasmid digested with *BsaA*I/*Hind*III to produce the targeting *APT* fragment containing the 35S::hygroR cassette (from pBHRF) flanked by genomic *PpAPT* sequences. Targeted integration of PpAPT-KO2 at the *APT* genomic locus confers resistance to 2-FA. We selected primary transformants (unstable + stable) with 25 mg/l hygromycin B (Duchefa). Integrative transformants were isolated following a second round of selection. Small pieces of protonemal tissue from these transformants were then transferred onto medium containing 5 μM of 2-FA to detect *APT* GT events. Experiments were repeated three times and statistically analysed using the *χ*^2^ test.

### Cytology

Binocular observations were made with a Nikon SMZ1000.

### Microscopy

4',6-diamidino-2-phenylindole (DAPI) staining of spore mother cells was adapted from the techniques described in (45,46). Leafy-shoots bearing immature sporangia were collected, fixed in 1:3 (v/v) acetic acid:ethanol and stored at 4°C.

For chromosome preparation by squashing one immature sporogonium was placed on a dry microscope slide in a drop of aceto-carmine stain (SIGMA, C6152) and crushed with a dissecting needle to remove the meiocytes and expose them to the stain. A coverslip was applied and meiocytes were squashed by tapping the coverslip gently with the flat back of a pencil. Slides were checked under a phase contrast microscope and slides showing meiocytes of the appropriate stages were immersed in liquid nitrogen until frozen (about 30 s), and their coverslips flicked with a razor blade. The air-dried preparation was dehydrated through an increasing ethanol series (70%–90%–100%, 2 min each). Air-dried slides were mounted in Vectashield antifade mounting medium (Vector laboratories H-1000) containing a mix of DAPI (Roche, 10236276001) and propidium iodide (Sigma, P4170), 2 μg/ml each.

For chromosome preparation by spreading fixed leafy-shoots bearing immature sporogonia were transferred to an embryo dish and washed in 10 mM citrate buffer pH 4.5 at room temperature and the buffer changed once before incubating with an enzyme mixture comprising 0.3% w/v cytohelicase (C1794), 0.3% w/v pectolyase (C8274) and 0.3% w/v cellulase (P5936) (all Sigma) in citrate buffer for 3.5 h at 37°C. Replacing the enzyme mixture with ice cold water stopped the reaction. A single sporogonium was transferred to a clean slide. The digested sporogonium was tapped out in 5 μl of water, using a fine needle. Note that 15 μl 60% acetic acid was added to the preparation and after gentle stirring with a hook, the slide was then placed on a 45°C heating block for 2 min. Air-dried slides were mounted in Vectashield antifade mounting medium (Vector laboratories H-1000) containing DAPI (Roche, 10236276001) at 2 μg/ml. All observations were made using a Zeiss Axio Imager2 microscope. All images were further processed with ZEN 2011 (Zeiss) or Adobe Photoshop 7.0.

## RESULTS

### Molecular and phenotypic characterization of the *PpRAD51B* deletion mutant

**Figure 1.**
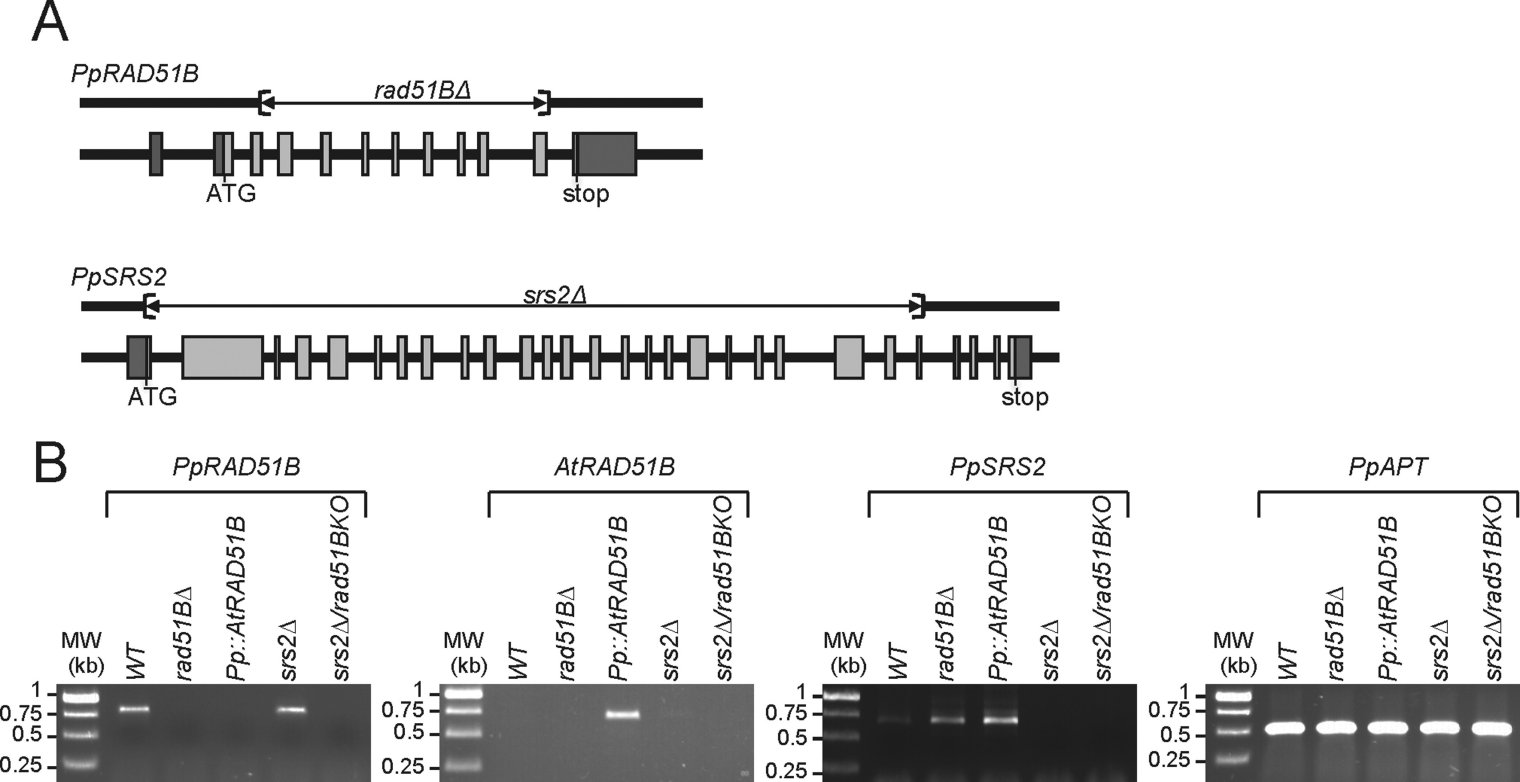
Structure and targeted disruption of Physcomitrella *RAD51B* and *SRS2* genes. (A) Structure of the *PpRAD51B* and *PpSRS2* genes. Exons are represented by shaded boxes, with 5′- and 3′-UTR sequences in darker grey. The region deleted by cre-lox excision of a selection cassette is shown as a line above each gene. (B) RT-PCR analysis of *RAD51B* and *SRS2* transcripts in WT and mutants plants. RNA was isolated from protonemal tissue of WT and mutants lines for cDNA synthesis and PCR amplification using gene-specific primers (PpRAD51B#19/#21; AtRAD51B#4/#5; PpSRS2#5/#6). The *PpAPT* transcript has been used as control (primers: PpAPT#14/#19). Primers are listed in Supplementary Table S1.

**Figure 2.**
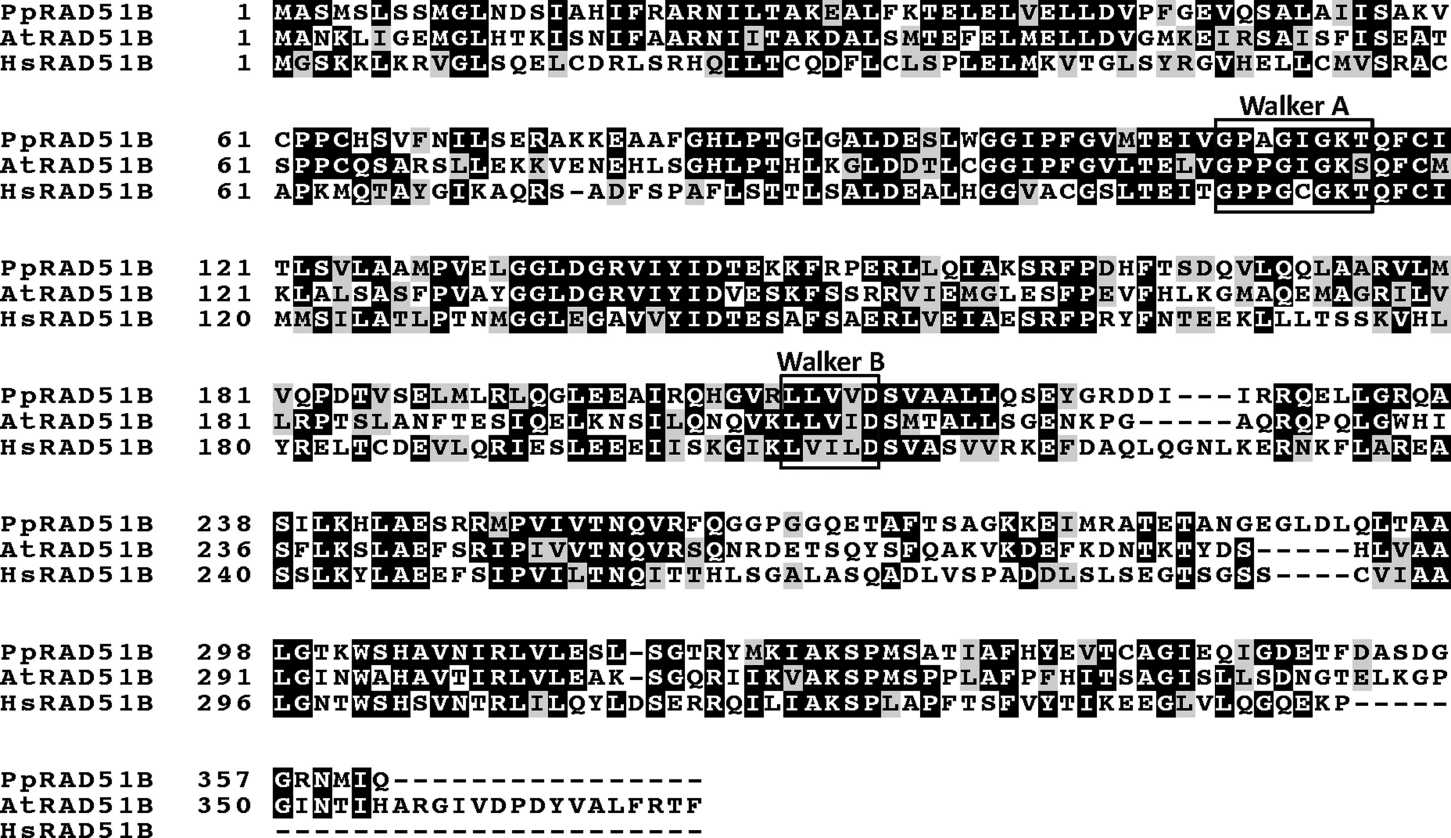
The Physcomitrella genome encodes a RAD51B homologue. ClustalW alignment of the RAD51B proteins from Physcomitrella,Arabidopsis and human. The amino acid sequences of the proteins are shown in the single-letter code. Gaps are indicated by dashes. Conserved amino acids are black shaded and similar amino acids are grey shaded. The positions of the amino acids in each protein are shown at left. Black boxes indicate the positions of Walker motifs A and B. Accession numbers used in this analysis are as follows: PpRad51B (Phpat.025G051900.1.p), AtRad51B (NP_180423) and HsRad51B (AAC39723).

Sequence homology searches of the Physcomitrella genome identified a single putative homologue for all the RAD51 paralogues except XRCC3 (Supplementary Figure S1). *PpRAD51B* is located on chromosome 25 (Phpat.025G051900) and our reverse transcriptase-PCR (RT-PCR) and Rapid Amplification of cDNA Ends (RACE) analyses of this gene, demonstrated that it consists of 12 exons and 11 introns (Figure 1A) and contains an open reading frame (ORF) of 362 amino acids (Figure 2). Phylogenetic analysis revealed that the protein encoded by *PpRAD51B* belongs effectively to the RAD51B family of proteins (Figure 2). The PpRAD51B protein has 46% and 36% sequence identity and 63% and 57% sequence similarity to AtRAD51B and HsRAD51B, respectively. There is extensive similarity among all the RAD51B polypeptides and PpRAD51B contained the characteristic features for RecA/Rad51 family proteins (Figure 2).

To investigate PpRAD51B function we generated a *rad51b* deletion mutant, named *rad51bΔ*, using targeted gene disruption followed by Cre/lox mediated elimination of the resistance cassette (Figure 1A and Materials and Methods). We confirmed that a portion of the coding region was removed by PCR using primers that flanked the deletion (Figure 1A) and sequence analysis confirmed the presence of a single LoxP site at the deletion site (not shown). RT-PCR analysis established that the full-length transcript was no longer produced in the mutant (Figure 1B). For all further experiments, we used two independent *rad51bΔ* strains, which displayed similar phenotypes.

### The PpRAD51B deletion mutants show subtle developmental defects

Like all mosses, the life cycle of Physcomitrella is characterized by an alternation of two generations, a haploid gametophyte that produces gametes and a diploid sporophyte where haploid spores are produced. A spore develops into a filamentous structure called protonema. Protonema filaments grow exclusively by tip growth of their apical cells and can originate side branches from subapical cells. Some side branch initial cells can differentiate into buds rather than side branches. These buds give rise to gametophores, more complex structures bearing leaf-like structures, rhizoids and the sexual organs: female archegonia and male antheridia. Sperm cells can swim from the antheridia to an archegonium and fertilize the egg within. The resulting diploid zygote originates a sporophyte, where thousands of haploid spores are produced by meiosis.

**Figure 3.**
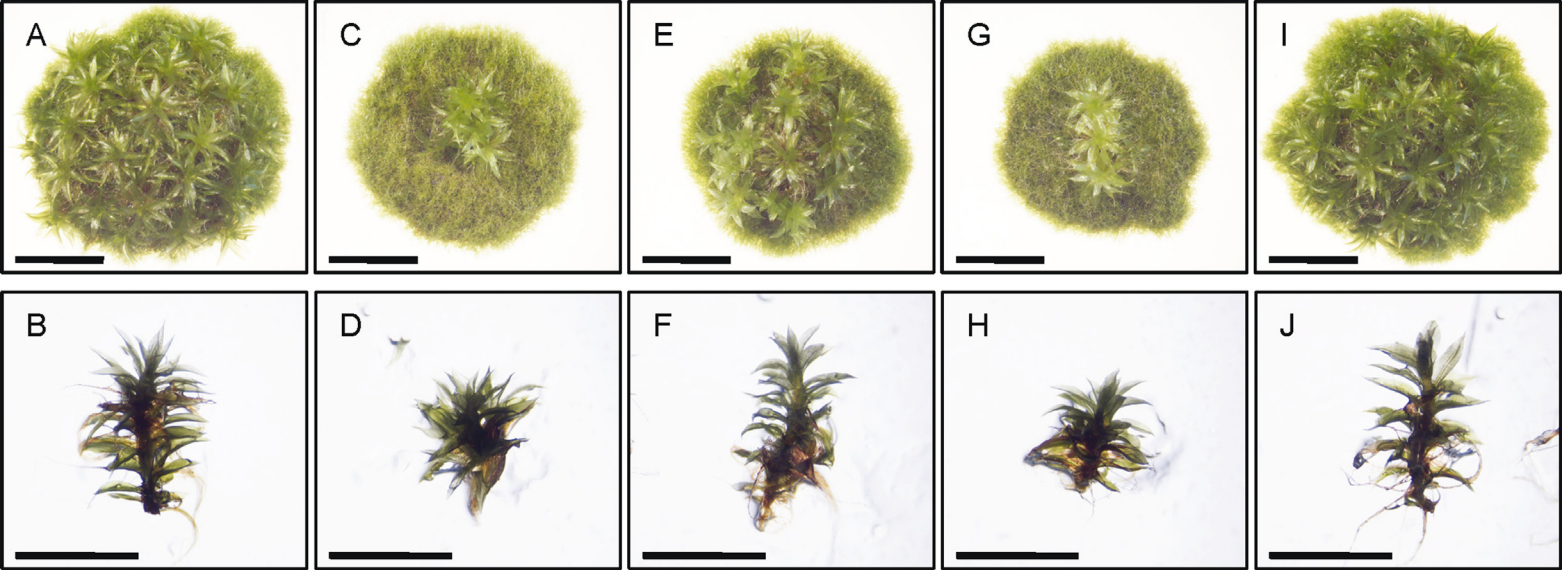
Vegetative development of the *rad51bΔ* mutants. Morphology of colonies and gametophores of WT (A, B), double rad51-1-2*?* mutant (C, D), two independent subclones of a *rad51bΔ* mutant (E, F and G, H) and srs2*?* mutant (I, J). The picture was taken after 30 days of growth. Scale bar = 5 mm.

The phenotype of the *rad51bΔ* mutants was characterized throughout this entire life cycle. By contrast with the double *rad51* mutant already described (41,47), that shows a strong developmental phenotype on rich medium (Figure 3C and Supplementary Figure S2), the *rad51bΔ* mutants display only a very weak defect in gametophytic development (Figure 3E and 3F), showing a normal protonema development but fewer gametophores than WT (81% of the WT, see Figure 3A and Supplementary Figure S2). However, after multiple subcultures we occasionally observed some individuals showing strong developmental phenotypes (see one example in Figure 3G and 3H) suggesting the possibility of a genetic instability in mutant lines.

**Figure 4.**
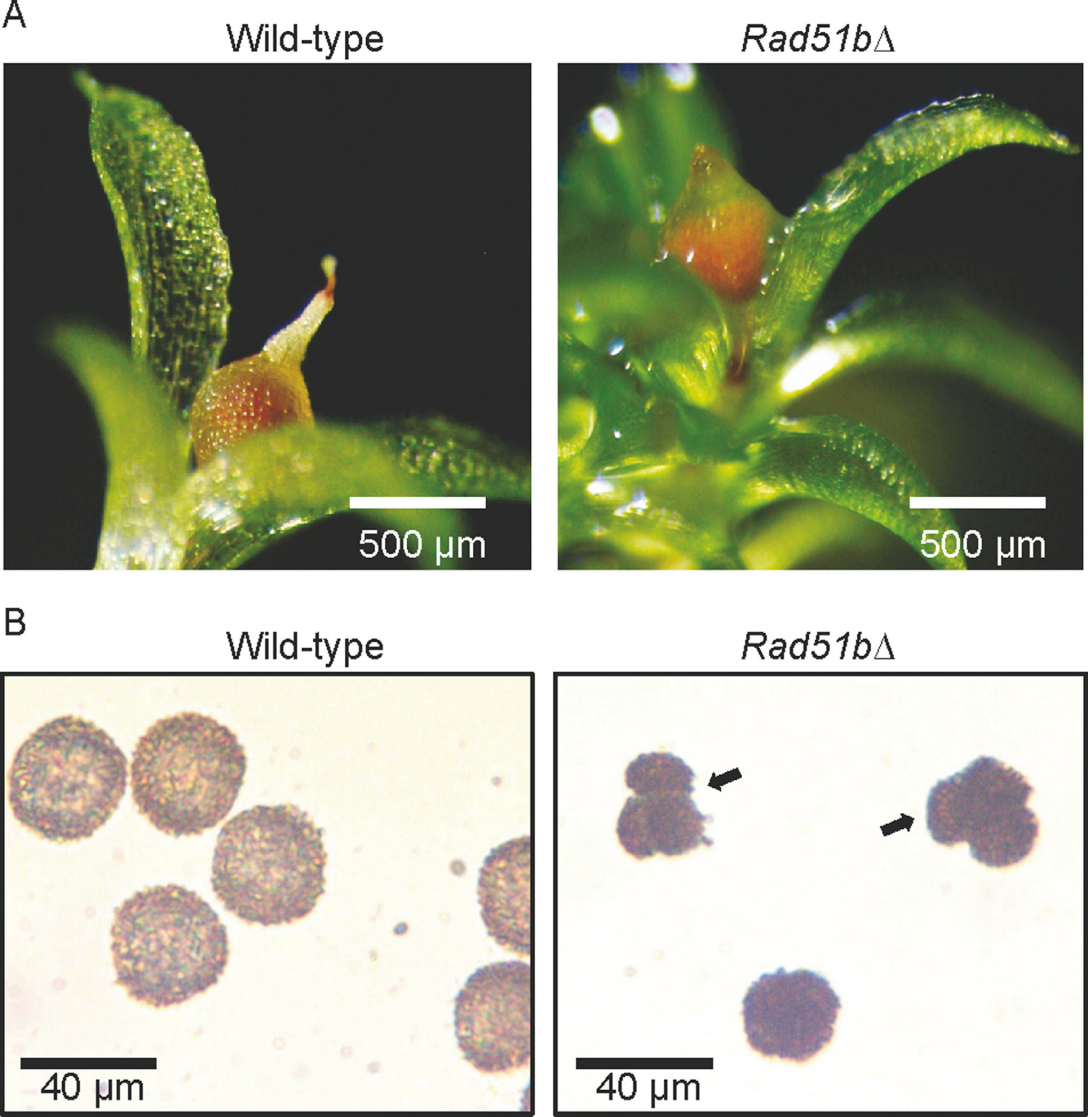
Phenotypes of Physcomitrella *rad51bΔ* mutant at later developmental stages. (A) WT and *rad51bΔ* gametophore and sporangium. (B) Spores obtained from mature WT, and *rad51bΔ* sporangium. Arrows indicate tetrad-like structures.

Gametophores produced by the *rad51bΔ* mutants are normal. However, compared to WT, *rad51bΔ* plants produce fewer mature sporophytes (Figure 4A) and these sporophytes produce capsules containing fewer spores (on average, 4000 spores/sporogonium for the WT and 900 spores/sporogonium for the mutant, *n* = 20). Spores produced by the *rad51bΔ* plants were smaller than the WT, irregular in shape, often remained as tetrads and could not germinate (Figure 4B and Supplementary Figure S3). Thus, if PpRAD51B is not essential for vegetative development, it is essential for fertility.

### The *rad51bΔ* mutants display a mutator phenotype

In order to confirm the genetic instability phenotype of the *rad51bΔ* mutant (see Figure 3G) and to evaluate its nature, we measured the intensity of its mutator phenotype. For this purpose, we investigated the effect of the loss of the *PpRAD51B* gene on mutation frequency of the *APT* reporter gene. Spontaneous mutations in this gene leading to loss of APT activity confer resistance to adenine analogues, such as 2-FA and can be selected on standard medium supplemented with 10 μM 2-FA. We tested a total of 5.3 × 10^6^ *rad51bΔ* protoplast derived colonies and 107.2 × 10^6^ WT colonies for resistance to 2-FA. We observed two resistant clones in the WT and a total of 145 2-FA resistant clones in the *rad51bΔ* mutant (Table 1). Thus, our analysis revealed that mutation rates were increased at least 1350 times in the *rad51bΔ* mutant as compared to WT, a value three times greater than the mutation rate observed in the *rad51-1-2Δ* double mutant (41) and 20-fold lower than that observed in the mismatch repair mutant *PpMsh2Δ* (40). In order to characterize the type of mutations found in the *rad51bΔ* mutant context we partially sequenced the *APT* gene of 13 randomly chosen 2-FA resistant clones (Supplementary Table S2). Mutations resulting from transitions, transversions and deletions were recovered. These data support a direct involvement of PpRAD51B in repairing naturally occurring DNA damage to prevent the accumulation of mutations in the genome.

**Table 1.** PpRAD51B is required to repair endogenous DNA damage

Genotype	Regenerants (×10^3^)	2-FA resistant	Rate in 10^6^
WT	107 200	2	0.02^a^
*rad51b?*	5300	145 (*P* = 3.9 × 10^−189^)^b^	27.3

^a^D.S. and F.N. unpublished, data from pooled WT controls of numerous mutator experiments.

^b^Differences were compared using Fisher's exact test.

### The *rad51bΔ* mutants are hypersensitive to UV-B and bleomycin C

**Figure 5.**
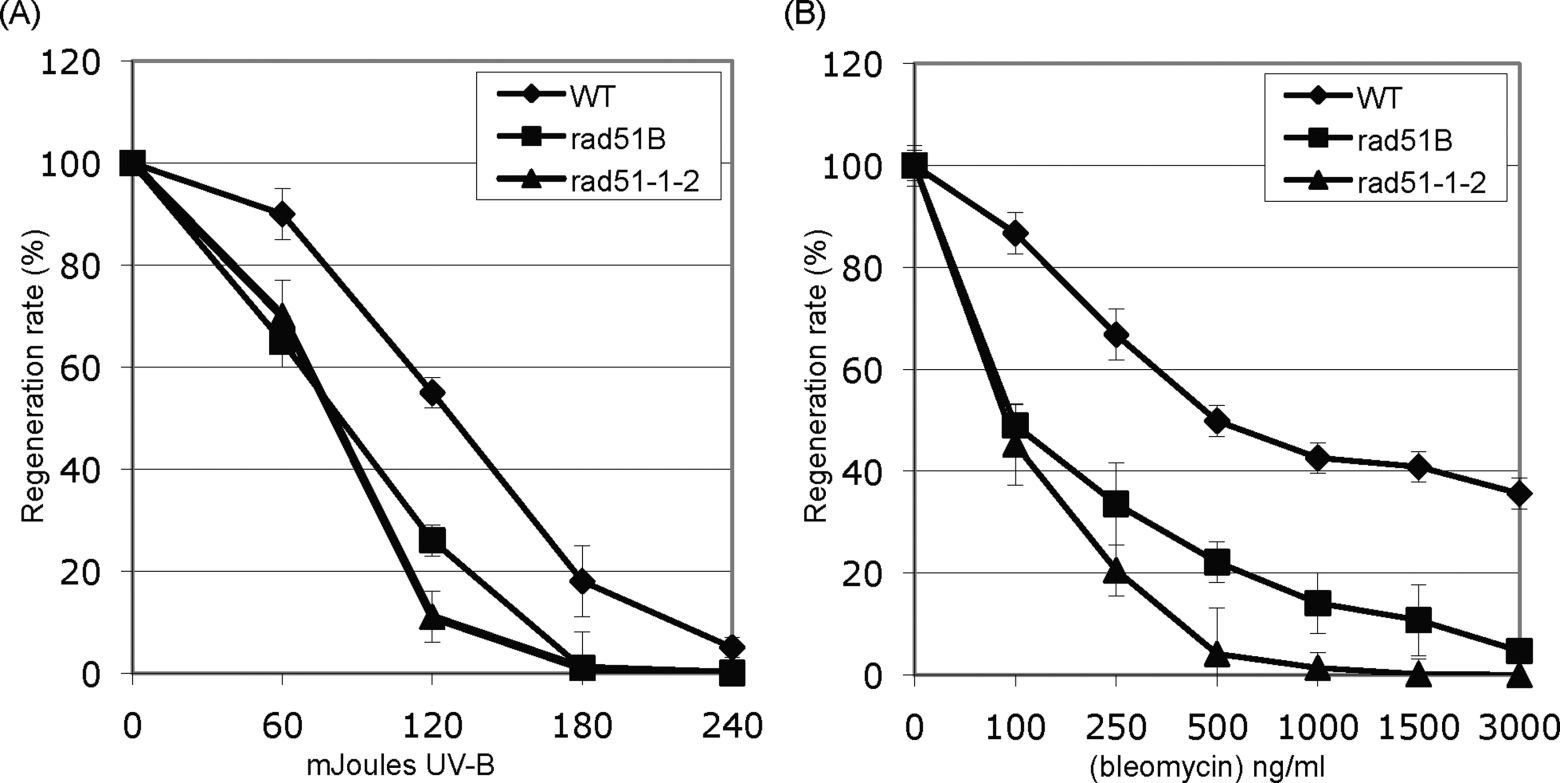
Hypersensitivity of the *rad51bΔ* and rad51-1-2*?* mutants to UV and bleomycin. Survival curves of WT, *rad51bΔ* and rad51-1-2*?* mutant protoplasts regenerating after exposure to UV (A) or bleomycin (B) treatments. Error bars indicate SD based on at least two independent experiments in all cases.

WT, *rad51-1-2Δ* and *rad51bΔ* mutant were also analysed for their sensitivity to DNA damaging agents. Sensitivity of mutants and WT strains to UV-B (308 nm) was investigated using a protoplast survival assay (40). The *rad51bΔ* mutant displayed increased sensitivity to UV-B compared to the WT; this increase is of the same order of magnitude as that observed for the *rad51-1-2Δ* mutant (Figure 5A). We further investigated sensitivity of the mutants to the DSB-inducing agent bleomycin (48). We tested the acute toxicity of bleomycin in WT and in the different mutants at the cellular level. Following incubation for 1 h with increasing concentrations of bleomycin, the ability of protoplasts to divide and regenerate into colonies was assessed by subculture on drug-free medium. Survival was calculated as the ratio of protoplasts surviving after 15 days of regeneration following treatment to the number of protoplasts undergoing normal regeneration without treatment. The LD50 for the WT was about 500 ng/ml bleomycin, whereas the *rad51-1-2Δ* and *rad51bΔ* cells were more sensitive, with an LD50 of 100 ng/ml (Figure 5B). The *rad51bΔ* cells show a sensitivity similar to the cells depleted of the *RAD51* genes, previously described as hypersensitive to bleomycin (44).

### GT is abolished in the *rad51bΔ* mutants

In order to investigate the involvement of the PpRAD51B protein in transgenesis, we quantified GT efficiency in WT and *rad51bΔ* mutant after transformation with linear pPpAPT-KO2 which confers hygromycin resistance (selective marker) and 2-FA resistance upon targeted integration at *PpAPT* locus, as described in (41). GT efficiency was determined as the frequency of 2-FA resistant plants among hygromycin-resistant integrative transformants. In the *rad51bΔ* mutant, GT was completely abolished, even though relative transformation frequencies (RTF for Hyg^R^ around 1.6 × 10^−3^) was similar to that observed in the WT strain with a homologous vector (Table 2). This experiment shows that PpRAD51B is essential for GT. Moreover, the fact that RTF is not altered in the *rad51bΔ* mutant suggests that the capacity of random integration of the linear vector is enhanced in the absence of PpRAD51B.

**Table 2.** Comparison of transformation and GT efficiencies

Genotypes	PpAPT-KO2				pBHRF	
	RTF^a^	Hyg^R^	2FA^R^	GT(%) ^b^	RTF^a^	Antib^R^
Wild type	1.2 ± 0.1^c^	294 (98 ± 16.5^c^)	202 (67.3 ± 8.0^c^)	69.1 ± 3.5^c^	0.15 ± 0.03^d^	35 (12 ± 4.6^c^)
*rad51bΔ*	1.6 ± 0.3^c^	360 (120 ± 16.1^c^)	0	0	1.21 ± 0.11^d^	267 (89 ± 11.5^c^)
*Pp::AtRAD51B*	1.4 ± 0.3^c^	312 (104 ± 6.2^c^)	0	0	nd	nd
*srs2Δ*	1.2 ± 0.2^c^	303 (101 ± 20^c^)	193 (64.3 ± 10.8^c^)	64 ± 3.2^c^	0.16 ± 0.05^d^	37 (12.3 ± 6.0^c^)
*rad51bΔ/ srs2Δ*	1.4 ± 0.2^c^	336 (112 ± 16.1^c^)	0	0	nd	nd

^a^RTF in % express the frequency of antibiotic resistant transgenic strains in the whole regenerated population.

^b^GT efficiencies (%) express the frequency of 2-FA resistant among the population of antibiotic resistant transgenic strains.

^c^Average and standard deviation was determined from three independent experiments.

^d^Average and standard deviation was determined from three independent experiments.

To further assess the specific role played by RAD51B in the mechanism of random DNA integration in Physcomitrella, we determined transformation efficiencies with a vector sharing no homology with the moss genome, pBHRF, in WT and *rad51bΔ* plants. We then compared these values with Hyg^R^ RTF achieved with the PpAPT-KO2 homologous vector. The absence of sequence homology on the transforming DNA reduces transformation frequencies ∼8-fold in WT (Table 2), which is consistent with previous reports (36). By contrast, Hyg^R^ RTF was similarly high with both vectors in *rad51bΔ* mutant, demonstrating that PpRAD51B directly represses random integration independently of the presence of moss homologous sequence on the transforming DNA. These data demonstrate that loss of function of RAD51B in Physcomitrella de-repressed the random integration that probably takes place via an illegitimate recombination pathway upon transformation as previously observed in the absence of RAD51 (41).

### A flowering plant RAD51B cannot complement the Physcomitrella *rad51bΔ* mutant defaults

GT efficiency is low in vascular plants compared to Physcomitrella but can be detected (49), implying that the recombination machinery necessary for GT is present. The Physcomitrella RAD51B is in the plant group of RAD51B proteins and is thus clearly of plant origin (Supplementary Figure S1). Furthermore, the Arabidopsis AtRAD51B protein shows a high level of identity (45%) with the PpRAD51B protein (Figure 2). In order to determine whether the AtRAD51B protein could fulfil the RAD51B function of Physcomitrella we replaced the *PpRAD51B* gene (ATG to stop) by the *AtRAD51B* cds (for details see Materials and Methods). Complete loss of the *PpRAD51B* transcripts and expression of the *AtRAD51B* cDNA were confirmed by RT-PCR in the Pp::AtRAD51B lines (Figure 1). For all experiments, we used two independent Pp::AtRAD51B lines; results were comparable in the two lines.

The Pp::AtRAD51B lines show the same developmental phenotypes as the *rad51bΔ* mutant (not shown) including sterility. GT efficiency was tested at the *PpAPT* locus as previously described. We observed that GT efficiency is completely abolished in both Pp::AtRAD51B lines compared to the WT (Table 1). These results indicate that expression of the Arabidopsis *RAD51B* cDNA under the control of the Physcomitrella *RAD51B* promoter is not able to restore a correct fertility or high GT efficiency.

### Absence of SRS2 does not restore GT in the *rad51bΔ* mutant

In *S. cerevisiae*, *Srs2* encodes a helicase which is a key regulator of Rad51 filament formation and disassembly and is thus considered as an ‘anti-recombinase’. It has been shown that deletion of *Srs2* can suppress the sensitivity to ionizing radiation of the mutants of the *S. cerevisiae* Rad51 paralogues, Rad55 and Rad57 (4,50). Moreover, the Rad55-Rad57 heterodimer has been shown to counteract the anti-recombination activity of the Srs2 helicase and a model has been proposed whereby Rad51 pre-synaptic filament formation is modulated by a balance between the stabilizing function of Rad55-Rad57 and the destabilizing function of Srs2 anti-recombinase (4). In order to test the possibility that the RAD51 pre-synaptic filament formation could be modulated by such a balance and antagonistic effects between the RAD51B and SRS2 proteins in Physcomitrella, we have produced a mutant in the *PpSRS2* gene and analysed its effect on GT.

By performing a sequence homology search with the yeast SRS2 protein sequence, we were able to identify only one candidate gene homologue in Physcomitrella, which is located on chromosome 1 (Phpat.001G116600). These results were in agreement with the previously described presence of an SRS2 homologue in bryophytes (51). Our RT-PCR and RACE analyses of this gene, named *PpSRS2*, demonstrated that it consists of 29 exons and 28 introns (Figure 1A), and contains an ORF of 1301 amino acids. Sequence analysis and domain determination revealed that the PpSRS2 protein has 50% and 26% sequence identity and 64% and 43% sequence similarity to AtSRS2 and ScSRS2, respectively, and that the conserved UVRD/REP domain of the proteins is clearly aligned (Supplementary Figure S4).

To investigate PpSRS2 function and its potential interaction with PpRAD51B we generated a *srs2* deletion mutant, named *srs2Δ*, and we retransformed this mutant to produce a double *srs2Δ/rad51b* mutant (Figure 1A). RT-PCR analysis established that the full-length transcripts were no longer produced in the mutants (Figure 1B). In terms of gametophytic development *srs2Δ* could not be distinguished from WT, nor *srs2Δ/rad51b* from *rad51bΔ* mutant (Figure 3). The *srs2Δ* mutant has normal sporogonia and produces normal and viable spores, the double *srs2Δ/rad51b* mutant, like the *rad51bΔ*, is completely sterile (Supplementary Figure S3).

We quantified GT efficiencies in the *srs2Δ* and *srs2Δ/rad51b* mutants after transformation with linear pPpAPT-KO2. In the *srs2Δ* mutant, GT efficiency was similar to the WT but in the double *srs2Δ/rad51b* mutant, as already observed in the simple *rad51bΔ* mutant, GT was completely abolished (Table 2). These data demonstrate that SRS2 is not involved in GT and that its loss of function does not restore GT in the *rad51bΔ* mutant context.

### Meiotic recombination is altered in the *rad51bΔ* mutant

In order to investigate the origin of the sterility of the *rad51bΔ* mutant we compared the spore germination efficiency between WT and the different mutants. The *rad51bΔ* mutant, *Pp::AtRAD51B* line and *srs2Δ/rad51b* double mutant produce no viable spores, compared to a mean germination efficiency of 97.6% and 96.2% for the WT and *srs2Δ* mutant plants, respectively (Supplementary Figure S3). Thus, PpRAD51B is essential for spore formation and loss of function of SRS2 or addition of the AtRAD51B function does not restore spore viability in the *rad51bΔ* mutant context. Spores are the direct product of meiosis. In order to explore the potential role of RAD51B in meiosis in Physcomitrella we have compared meiotic progression between the WT and the *rad51bΔ* mutant.

**Figure 6.**
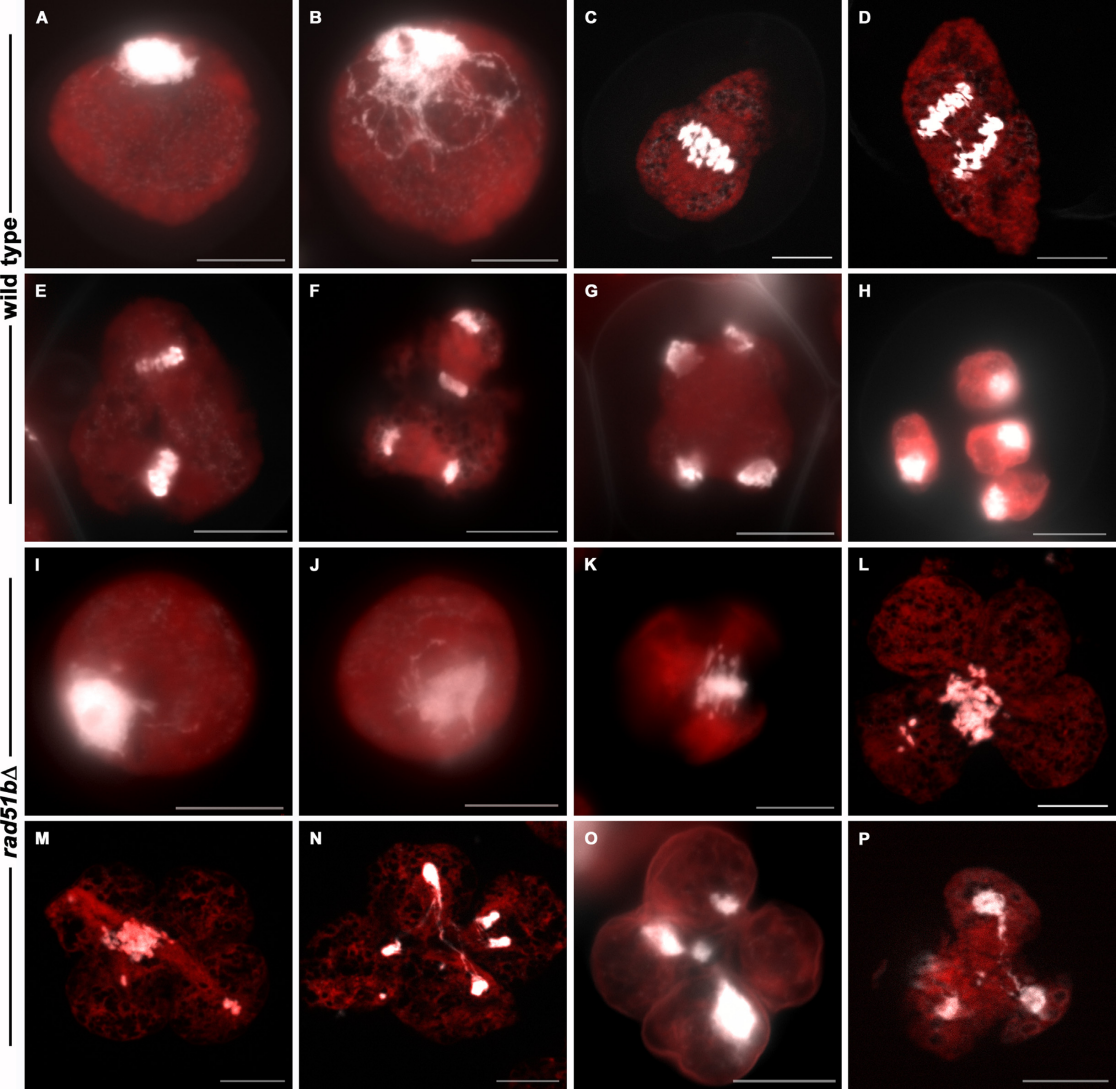
DAPI staining of WT and *rad51bΔ* meiotic chromosomes. (A, B) WT and (I, J) *rad51bΔ* prophase I, (C) WT and (K) *rad51bΔ* metaphase I, (D) WT and (L) *rad51bΔ* anaphase I, (E) WT and (M) *rad51bΔ* metaphase II, (F) WT and (N) *rad51bΔ* anaphase II, (G) WT and (O) *rad51bΔ* telophase II, (H) WT mature spore and (P) *rad51bΔ* unseparated spores. Scale bar = 10 μm.

Young sporangia were first examined by fluorescence microscopy after cell squashing and DAPI staining of the chromosomes. In the WT (Figure 6), the Physcomitrella chromosomes condense during meiotic prophase I (Figure 6A and B) and can be seen as 27 pairs of homologous chromosomes (bivalents) in metaphase I (Figure 6C and Supplementary Figure S5). Homologous chromosomes then separate from each other and migrate to the opposite poles of the cell in anaphase I (Figure 6D). The second meiotic division starts with the alignment of chromosomes in metaphase II (Figure 6E) and is followed by separation of sister chromatids in anaphase II (Figure 6F). Telophase II ensues, chromosomes decondense (Figure 6G) and cytoplasm is partitioned to produce a tetrad containing four haploid microspores (Figure 6H), which will differentiate into mature spores (Figure 4B). In young sporangia of the *rad51bΔ* mutant, prophase I proceeds (Figure 6I and J) but, at the transition between metaphase I and anaphase I, a mass of entangled chromosomes can be seen in place of the 27 expected bivalents (Figure 6K). Hardly any chromosome segregation could be observed, but cytokinesis nevertheless takes place, always generating four daughter cells with aberrant quantities of DNA (Figure 6L–N) and eventually producing unbalanced tetrads (Figure 6O and P). Maturation of these aberrant tetrads of microspores results in aggregated spores with abnormal shape (Figure 4B).

**Figure 7.**
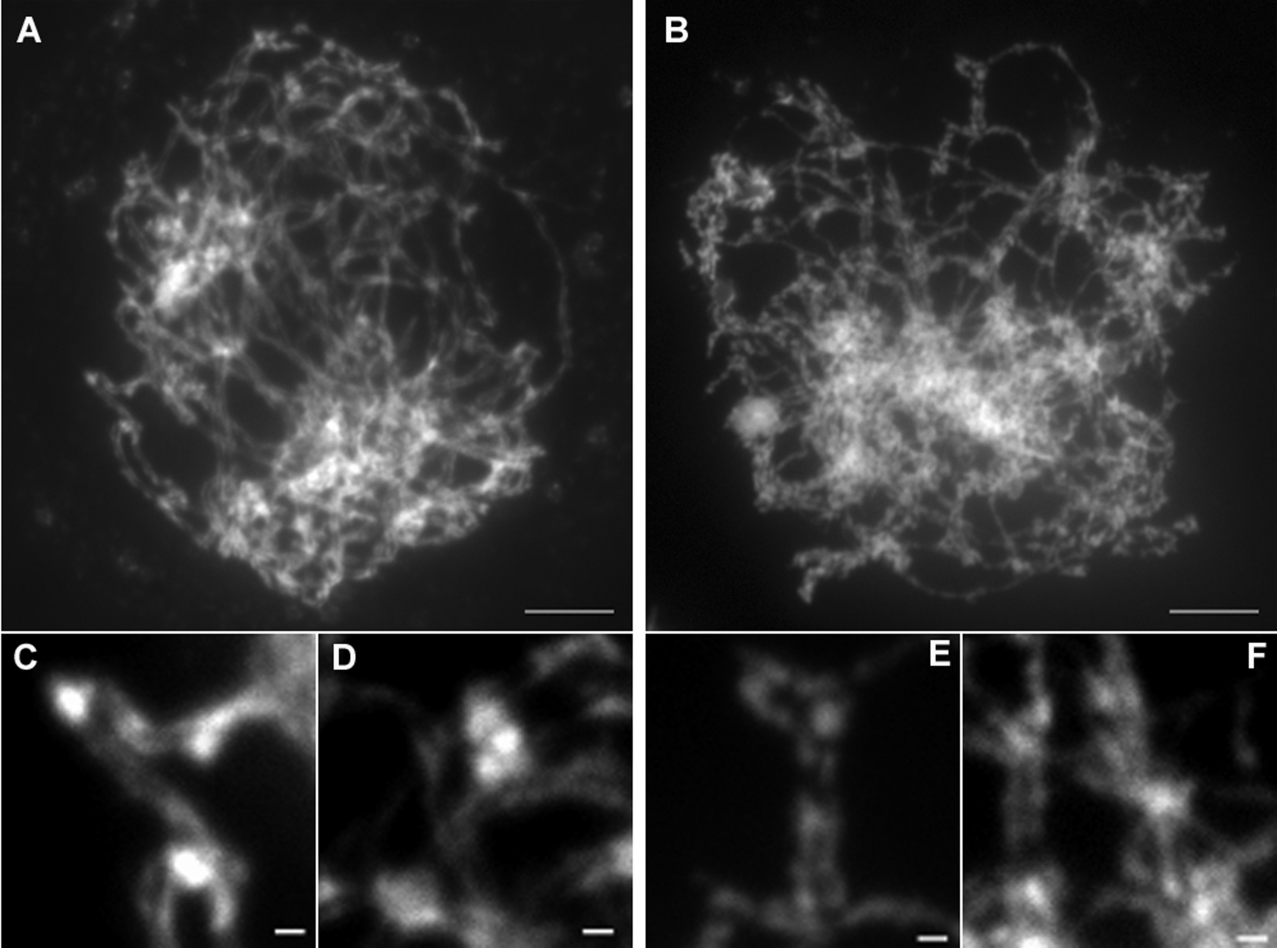
Spread analysis of WT and *rad51bΔ* mutant prophase. (A, C, D) WT and (B, E, F) *rad51bΔ* mutant chromosome spreads (pachytene) counterstained with DAPI. C, D and E, F are enlarged regions of A and B, respectively. Scale bar = 5 μm for A and B and 1 μm for C, D, E and F.

In order to better characterize meiotic prophase, we established a chromosome spreading technique (see Materials and Methods) that allowed the visualization of the close association of chromosomes along their length (Figure 7A, C and D) (*n* = 25). These close associations are very likely to reflect the formation of mature synaptonemal complexes between homologous chromosomes during WT meiosis (pachytene stage). In the *rad51bΔ* mutant, such close association was never observed (Figure 7B) (*n* = 19), except on short portion of the nucleus (Figure 7E and F).

Taken together, these results show that RAD51B in Physcomitrella is required for early meiotic events and for the formation of normal bivalents.

## DISCUSSION

We report here the identification and characterization of the Physcomitrella mutant for the *RAD51* paralogue *RAD51B*. The first important result of this study is that *Pprad51b* mutants display only a weak developmental phenotype, characterized by a slight reduction of gametophore production. This is in strong contrast with the situation in vertebrates where mutation in RAD51B impairs viability (5), and also different from vascular plants were the *rad51b* mutant does not show any developmental phenotype (22).

Although *Pprad51B* mutants are viable and show no strong phenotypic defect under normal conditions, these mutants show an increase in sensitivity to UV-B compared with the WT. A role for RAD51B in UV-C-induced apoptosis of hamster CHO cells has been proposed (52), and more recently, a stronger increase of UV-C-induced HR frequency has been observed in the Arabidopsis *rad51b* mutant compared to the WT (35). However, the role of the RAD51B protein in mid-wavelength UV (UV-B) sensitivity, that is likely to be more biologically relevant than UV-C, has not been studied and we show here for the first time the involvement of RAD51B in this stress response. Deciphering the UV-B resistance pathway in plants is of particular interest in light of the potential influence of the solar UV-B radiation on the genomic stability of plant populations (53).

The *Pprad51B* mutant also shows a strong increase in sensitivity to the DSB inducing agent bleomycin. Hypersensitivity to bleomycin is a hallmark of mutants affected in RAD51 and the RAD51 paralogues in *S. cerevisiae* (54) and can be correlated to a default in HR-dependent DNA DSB repair. A *Rad51b* mutant of DT40 chicken cells shows a slight increase in sensitivity to gamma rays, another DSB inducing agent (14). In vascular plants, the *rad51b* mutant of Arabidopsis shows a normal or only a slight increase in sensitivity to gamma rays (21,22) and is slightly more sensitive to bleomycin than the WT (23). The strong increase of sensitivity to bleomycin of the Physcomitrella mutant is in good agreement with an essential role of the HR-dependent DNA DSB repair pathway in this moss (41), and confirms, in regard of the difference of sensitivity to bleomycin between the Physcomitrella and Arabidopsis *rad51b* mutants, the differences in the use of recombination pathways between the two plants (47).

We have shown previously that the loss of RAD51, that is essential for HR function, induces genetic instability in Physcomitrella (41). Here, we show that the *rad51b* mutant also exhibits a mutator phenotype and thus that the RAD51B protein is also essential for genetic stability. The higher frequency of mutations in the *rad51* and *rad51b* mutants compared to the WT is probably due to the role of the HR machinery in error-free-mediated damage repair during DNA replication blocks (2,55). However, it must be noted that, in addition to its general role in fork-associated DNA damage repair via HR, RAD51 has also an early role during replication blocks, facilitating replication fork restart when forks are still viable (56,57). In animal cells, this second RAD51 function consists, in part, of a direct interaction between polEta and RAD51 that is essential for the restart of the replication fork (58). If this second role of RAD51 is conserved in Physcomitrella, failure to restore synthesis and replication in the absence of RAD51 (15,55,58) could explain the decrease in growth rate observed in the Physcomitrella *rad51* mutant and its associated developmental phenotype (41,47). This second function of RAD51 would be independent of RAD51B and in the *rad51b* mutant, the presence of RAD51 and polEta could permit the restart of the replication fork and thus cell division would be only slightly affected in this context. Nevertheless, in addition to the mutations resulting from the failure to repair DNA damage via HR, the *rad51b* mutant would accumulate mutations due to the restart of replication via an error-prone polymerase (59). Supporting this hypothesis, mutations, like deletions, that were not found in the Physcomitrella *msh2Δ* mutant context for example (40), and could result from the translesion synthesis-mediated bypass of damages (60) can be found in the *rad51bΔ* mutant. Taken together, these results could account for the weaker developmental phenotype and stronger mutator phenotype observed for the Physcomitrella *rad51b* mutant compared to the *rad51* mutant.

The lethality of the RAD51B mutations in animals has made the study of the role of RAD51B in HR difficult. Moreover, due to the absence of an efficient test for GT in most animals and vascular plants, no data are available on the putative role of RAD51B in HR-mediated GT in these organisms. However, studies in human cells recently showed that RAD51-dependent HR is reduced, but not abolished, in the absence of RAD51B (61). Similarly, based on the use of an *in planta* intrachromosomal recombination assay, it has been shown in Arabidopsis that somatic recombination is decreased but not abolished, in a *rad51b* mutant context suggesting that RAD51B is involved but not essential for RAD51 nucleofilament formation in Arabidopsis (24). By contrast, RAD51B has been shown to be essential for HR-mediated GT in chicken DT-40 cells (14). The situation is also different in unicellular eukaryotes: while RAD51B is essential for GT in trypanosomatidae (62), the Rad55 and Rad57 paralogues are not essential for GT in *S. cerevisiae* (63). We show here that the RAD51B protein is absolutely required for HR-mediated GT in Physcomitrella. The essential role of this RAD51 paralogue in HR for GT competent eukaryotes, such as Physcomitrella, trypanosomatidae and DT-40 cell lines contrasts with the less prominent role of the RAD51 paralogues for GT observed in *S. cerevisiae* (63). The importance of RAD51B for GT in Physcomitrella clearly distinguishes these two species and reflects the more important role of RAD51 in GT in Physcomitrella compared to *S. cerevisiae* (41).

The HR machinery is essential for targeted integration of a homologous vector. Does this mechanism affect untargeted integration of a non-homologous vector? In *S. cerevisiae*, illegitimate recombination of non-homologous DNA fragments occurs at low frequencies (64), and the absence of RAD51 leads to a further decrease in the number of Illegitimate Recombination (IR) events compared to WT (63). By contrast, in *A. nidulans*, *S. pombe* and Physcomitrella (41,65–66) the absence of RAD51 increases IR rates. Our data show that in Physcomitrella, deletion of *RAD51B*, like deletion of *RAD51*, leads to an increase in IR. This implies that whereas in *S. cerevisiae* the HR machinery is necessary for the integration of exogenous DNA with or without homology, in moss there is clearly competition for DNA integration between HR and IR (probably NHEJ). Thus, the idea that the outcome of transgenesis reflects the balance between HR- and IR-mediated DNA repair is strongly supported in moss.

The Rad55/Rad57 complex promotes Rad51 nucleoprotein filament stabilization and facilitates Rad51-dependent strand invasion reactions in yeast (2). Because the mammalian RAD51 paralogue complexes share biochemical features and genetic functions with the Rad55/Rad57 heterodimeric complex it has been proposed that early role of the RAD51 paralogues in HR is to promote RAD51 nucleoprotein filament assembly. Recently, *in vitro* studies have shown that the RAD51B-RAD51C complex can regulate the stability of the RAD51 nucleoprotein filament by acting as a pro-recombinase against the BLM helicase that acts as an anti-recombinogenic factor (18). However, evidence of direct *in vivo* interaction between RAD51 and the RAD51 paralogues is still lacking and the respective roles of the different RAD51 paralogues in the formation of the RAD51 nucleofilament is still unclear (6). In *S. cerevisiae*, the Rad55/Rad57 paralogue-mediated Rad51 filament assembly was shown to be counterbalanced by the Srs2 anti-recombinase (4,50) which led us to test the potential interplay between SRS2 and RAD51B in Physcomitrella. For this purpose we isolated the first plant *srs2* mutant. Our results show that the *srs2* mutant has no obvious developmental phenotype, is fully fertile, is not affected in GT and shows a meiotic behaviour similar to WT which is very different from the situation in yeast where Srs2 is involved in somatic DNA repair and in meiosis (67). We also observed that the absence of *SRS2* does not restore the GT capacity of the *rad51b* mutant. It should be noted, however, that even in yeast, *rad55* or *rad57* recombination defects are only partially restored by the *srs2* mutation (4). The diversification of the RAD51 paralogues in multicellular eukaryotes probably reflects a specialization of these paralogues in different aspects of recombination. One possibility is that RAD51B is not involved in the recombination steps potentially controlled by SRS2 and that another RAD51 paralogue might interact with SRS2 to control RAD51 loading in multicellular eukaryotes, a hypothesis that we are currently testing.

Yeast mutants impaired in any of the RAD51 paralogues are defective in meiotic recombination and exhibit reduced spore viability (3). The situation in multicellular eukaryotes is more complex. Indeed, while the XRRC3 and RAD51C paralogues have been shown to be indispensable for meiosis in *Drosophila*, Arabidopsis and rice (25–27,68–69), the RAD51D paralogue has been shown to be essential for meiosis only in rice (28). Moreover, it was recently shown in Arabidopsis that the absence of XRCC2, and to a lesser extent of RAD51B, increases meiotic crossing over rates (24). By contrast, we show here that RAD51B is essential for meiosis in Physcomitrella and that in its absence, homologous chromosomes do not form bivalents at metaphase I but instead appear as a mass of entangled chromosomes that cannot subsequently segregate. This phenotype is associated with evidence of synapsis defects during prophase and is reminiscent of the meiotic phenotype of the flowering plant mutants affected in meiotic DSB repair, such as *rad51*, *xrcc3*, *mnd1* or *hop2* (29). Therefore, it is very likely that RAD51B is involved in meiotic recombination in Physcomitrella, although more studies will be necessary to define precisely at which steps of meiotic DSB repair. Further studies will also be necessary to determine whether the essential role of RAD51B during meiosis can be extrapolated to other organisms. The high expression levels of RAD51B observed in mouse testis (19) suggests that this could be the case.

Our results and the recent demonstration that RAD51D is also essential for rice meiosis (28) contrast strikingly with the role of these genes during Arabidopsis meiosis (24) and reinforce the idea that the function of the RAD51 paralogues during meiosis can vary from one species to another. More generally, the differences observed between the Physcomitrella and Arabidopsis *rad51b* or *rad51* mutants and the fact that the Arabidopsis *RAD51B* or *RAD51* genes cannot complement the defects observed in the corresponding Physcomitrella mutants (this study and unpublished data), leave the open question of whether there are distinctly different somatic and meiotic HR machineries in bryophytes and vascular plants.

## SUPPLEMENTARY DATA

Supplementary Data are available at NAR Online.

SUPPLEMENTARY DATA
