# Supplementary material for: RAD51B plays an essential role during somatic and meiotic recombination in Physcomitrella
Source: Nucleic Acids Res. 2014 Sep 26;42(19):11965–78. doi: 10.1093/nar/gku890 (PMC4231755; doi:10.1093/nar/gku890)
Supplement: SUPPLEMENTARY DATA [file supp_42_19_11965__index.html]

RAD51B plays an essential role during somatic and meiotic recombination in Physcomitrella — RAD51B plays an essential role during somatic and meiotic recombination in Physcomitrella — SUPPLEMENTARY DATA 

# RAD51B plays an essential role during somatic and meiotic recombination in Physcomitrella

## SUPPLEMENTARY DATA

**Files in this Data Supplement:**

- SUPPLEMENTARY DATA
