# Supplementary material for: RAD51B plays an essential role during somatic and meiotic recombination in Physcomitrella
Source: Nucleic Acids Res. 2014 Sep 26;42(19):11965–78. doi: 10.1093/nar/gku890 (PMC4231755; doi:10.1093/nar/gku890)
Supplement: SUPPLEMENTARY DATA [file supp_gku890_nar-01357-d-2014-File009.pdf]

Supplemental Table 1. Primers used in this study.

Locus	Primer name	orientation	Sequence 5'-3'
RAD51B	PpRAD51B#1	Forward	ATTGTGGTGCCTTGTGTGTATTGT
	PpRAD51B#2	Reverse	CCGTGTGCTTCCTGCTACTGA
	PpRAD51B#19	Forward	AATCCGCACTCGCTATCATCA
	PpRAD51B#21	Reverse	CGAGGCGGATGTTGACTG
	AtRAD51B#4	Forward	CATGAAACTTGCGTTATCAGCTT
	AtRAD51B#5	Reverse	CAGTCCCGTTGTCGCTCAG
SRS2	PpSRS2#5	Forward	GCCCATATCCATCGTCTCCTT
	PpSRS2#6	Reverse	GAGTAAAGCCGTAGAGCACAG
	PpSRS2#10	Forward	GCAAGTTCCAAAGGCCTCCTAT
	PpSRS2#11	Reverse	GAGCCTTGTTTGCTTTGGTTAC
APT	PpAPT#14	Forward	AGATGTCGGCCTCCAAGGATG
	PpAPT#16	Forward	CCACCCATTGCTCTTGCCATC
	PpAPT#19	Reverse	CCCGACAACTTCTCACGACCC
	PpAPT#2	Forward	TTTTTGCGCTCGCTGTTTCTG
	PpAPT#20	Reverse	TAAATAATTCTGACCCAAAGT

Supplemental Table 2.

Mutations identified in the *APT* genomic sequence in *rad51b*Δ2-FA resistant clones

Clone #	Genomic position[a]	Type of mutation	consequence[b]
20	2185G>A	transition	130Asp>Asn
23	2754G>A	transition	defect in intron 5 splicing
31	2182G>A	transition	129Asp>Asn
6	1530C>G	transversion	premature stop codon
19	1763G>T	transversion	90Lys>Asn
22	2146G>T	transversion	premature stop codon
28	2449A>T	transversion	defect in intron 4 splicing
5	1530C>A, 1531C>T	transversion, transition	premature stop codon
10	1770delT	deletion (1bp)	premature stop codon
2	2503_2553del	deletion (51 bp)	defect in intron 5 splicing
4	2095_2133del	deletion (39 bp)	Pro97_Cys111del
25	1211_1568delinsAGATGTATGATGA	deletion/insertion	deletion of Exon1
30	1564_1829delinsGACGTCATTG	deletion/insertion	deletion of Exon3

[a] Position 1 corresponds to the first nucleotide in the genomic *PpAPT* sequence DQ117987

[b] Position 1 corresponds to the ATG codon in the PpAPT protein.

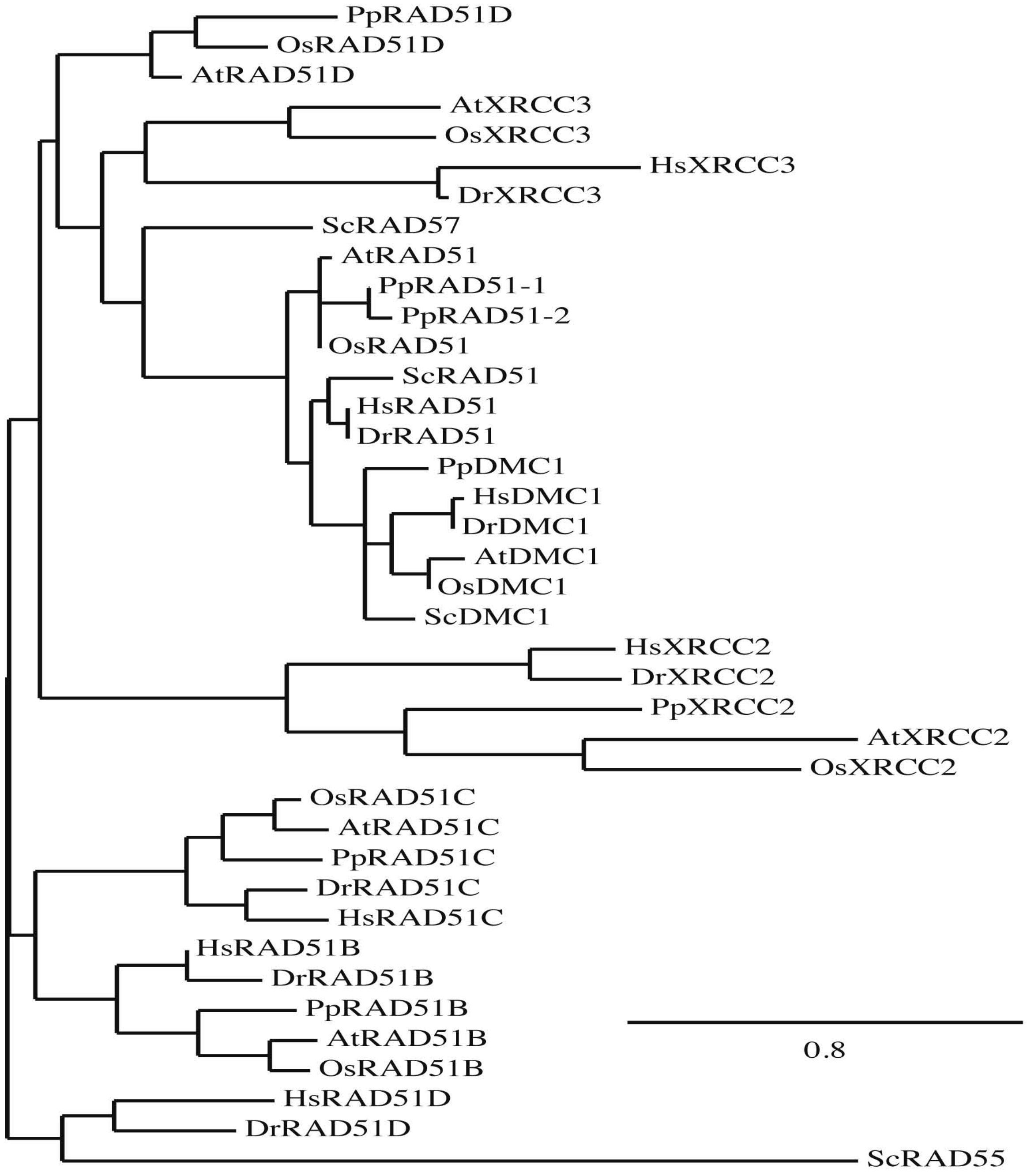

Figure S1 Physcomitrella genome encodes homologs of the five Rad51 paralogs

The dendrogram illustrates the sequence relationships among 39 Rad51-like proteins in Saccharomyces cerevisiae (Sc), Danio rerio (Dr), Homo sapiens (Hs), Oryza sativa (Os), Arabidopsis thaliana (At) and Physcomitrella patens (Pp). The branch lengths are proportional to the sequence divergence. The scale represents 0.8 substitutions per site.

Accession numbers for 37 deduced amino acids sequences used in this analysis are as follows: PpDmc1 (Phpat.020G030700.2.p), PpRad51-1 (CAC86603), PpRad51-2 (CAC86604), PpRad51B (Phpat. 025G051900.1.p), PpRad51C (Phpat.005G047200.1.p), PpRad51D (Phpat.023G036100.1.p), PpXrcc2 (Phpat. 001G024300.3.p), AtDmc1 (AAC49617), AtRad51 (CAA04529), AtRad51B (NP_180423), AtRad51C (BAB64343), AtRad51D (NP_172254), AtXrcc2 (NP_851268), AtXrcc3 (BAB64342), DmCG2412 (NP_610466), DmCG6318 (NP_573302), DmRad51 (BAA04580), DmSpn-B (NP_476740), DmSpn-D (AAP13056), HsDmc1(BAA10970), HsRad51 (BAA02962), HsRad51B (AAC39723), HsRad51C (AAC39604), HsRad51D (AAC39719), HsXrcc2 (CAA70065), HsXrcc3 (AAC05368), ScDmc1 (AAA34571), ScRad51 (BAA00913), ScRad55 (AAA19688), ScRad57 (AAA34950).

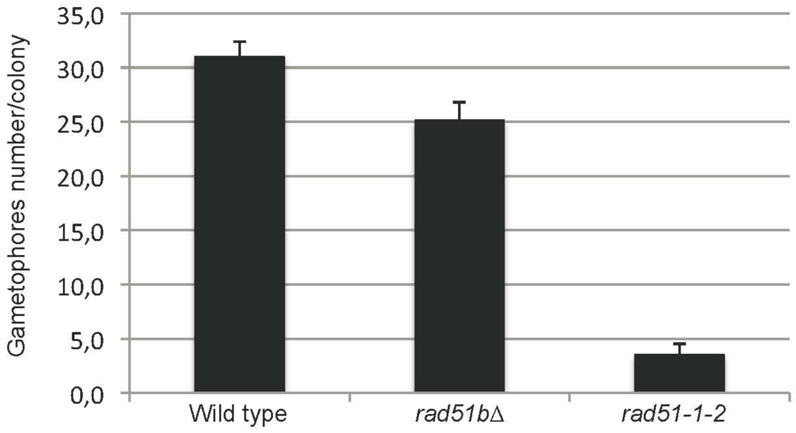

Figure S2. Gametophores number per colony for wild type *rad51b*Δ *and rad51-1-2*.
Gametophores number has been counted for 8 individual colonies for each genotypes. Colonies were 30 days old.

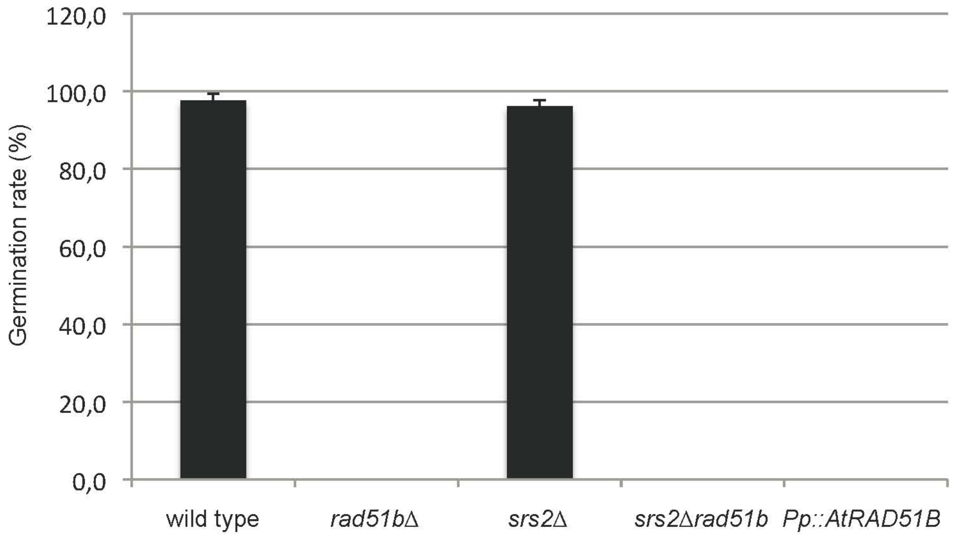

Figure S3. Germination rate of spores obtained from wild type*, rad51bΔ, srs2Δ, srs2Δrad51b* and *Pp::AtRAD51B* capsules.

Freshly harvested spores from wild type*, rad51bΔ, srs2Δ, srs2Δrad51b* and *Pp::AtRAD51B* capsules were sown on PpNH4 medium. Percentages of germination were calculated three days after sowing. Error bars indicate SD based on three independent experiments.

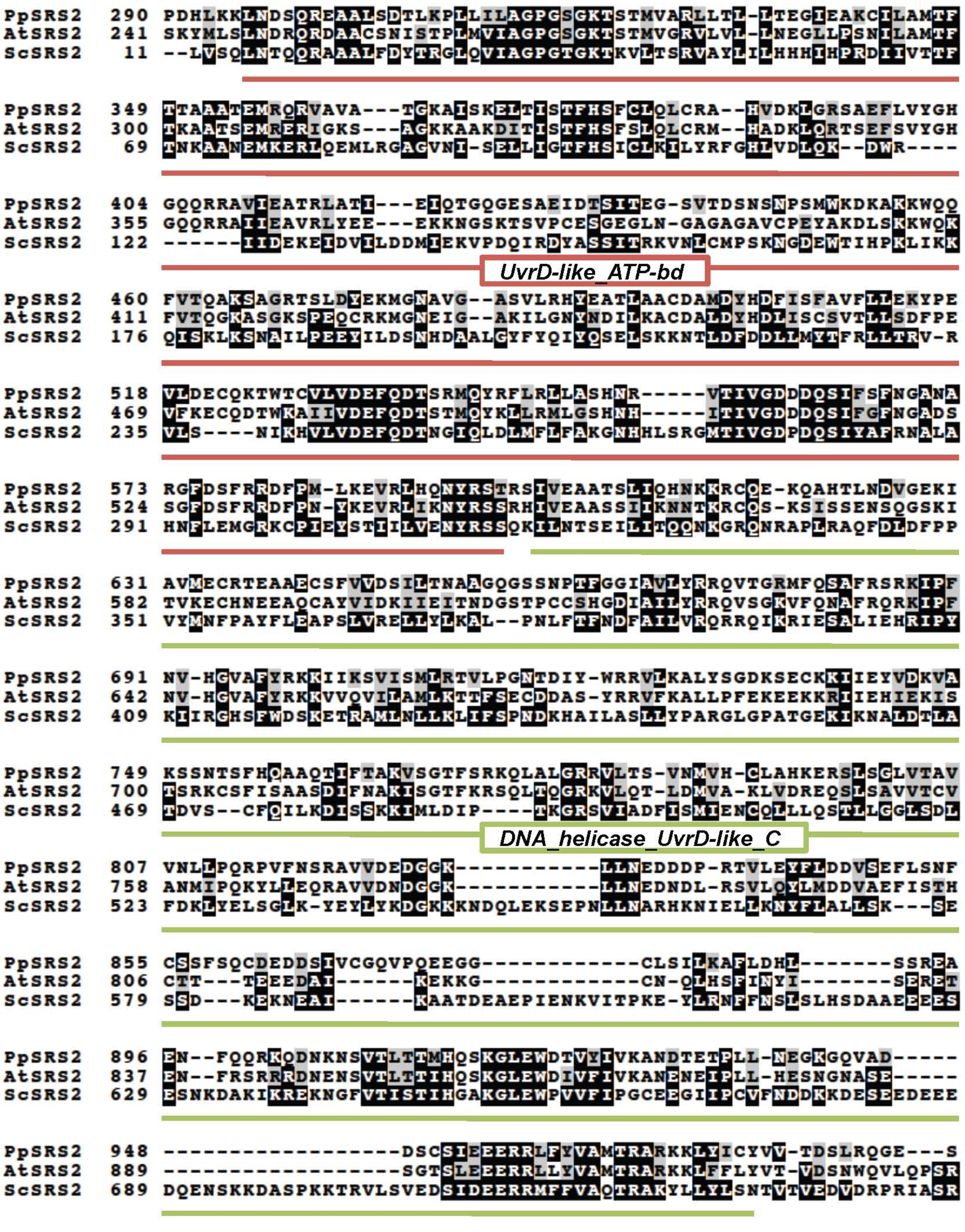

Figure S4. Physcomitrella genome encodes a SRS2 homolog.
The UVRD domain of PpSRS2 is shown in a ClustalW alignment with SRS2 of *A. thaliana* and *S. cerevisiae*. According to the InterProScan tool (http://www.ebi.ac.uk/Tools/InterProScan/), the sequence of the UVRD-domain ranges from amino acid 296 to 981 from PpSRS2 (IPR000212, DNA helicase, UvrD/REP type) and contains two Interpro domains. IPR014016 (UvrD-like Helicase, ATP-binding domain) ranges from aa 296 to 582 and IPR014017 (DNA helicase, UvrD-like, C-terminal) from aa 589 to 975. The amino acid sequences of the proteins are shown in the single-letter code. Gaps are indicated by dashes. Conserved amino acids are black shaded and similar amino acids are gray shaded. The positions of the amino acids in each protein are shown at left. Accession numbers used in this analysis are as follows: PpSRS2 (Phpat.001G116600.1p), AtSRS2 (ACY64665) and ScSRS2 (EEU06720).

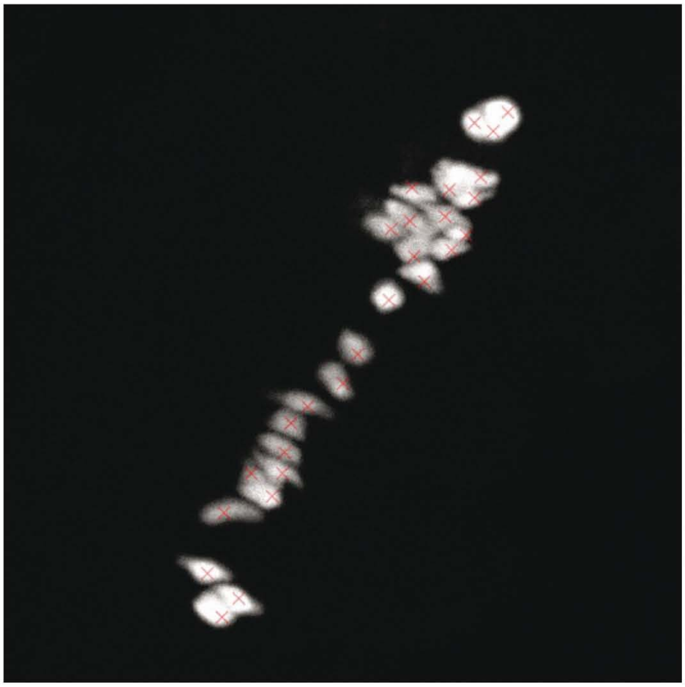

Figure S5. Counting of bivalents during metaphase I. Wild type young sporogone were examined by fluorescence microscopy after cell squashing and DAPI staining of the chromosomes. 27 bivalents can be observed.
